# Comparing reliability between 3D imaging and 2D photography for external nasal anthropometry

**DOI:** 10.1038/s41598-022-08714-y

**Published:** 2022-03-16

**Authors:** Yoon-Soo Seo, Ki-Hun Jo, Joo-Yeon Kim, Jae-Hwan Kwon

**Affiliations:** Department of Otolaryngology-Head and Neck Surgery, Kosin University College of Medicine, Seo Gu Am-nam dong, Busan, 602-702 Korea

**Keywords:** Anatomy, Imaging and sensing

## Abstract

This study investigates and compares the reliability and reproducibility of two facial anthropometric methods about external nasal angles, 3D imaging and conventional 2D photography. 2D photograph images and 3D images about external nose of 30 volunteers were taken using digital camera and Morpheus 3D scanner. To evaluate intra-rater reliability, each images were taken over two different days for each subject by the same researcher. To evaluate inter-rater reliability, another researcher took each images for each subject on the first day. The reliability of each method for measuring 4 external nasal angle is obtained using intraclass correlation coefficient (ICC) and compared. Inter-rater and intra-rater reliability of both 3D imaging and 2D photography had excellent agreement in all 4 nasal angles. In the nasofacial angular parameter, Inter-rater ICC, 2D photography was significantly higher than 3D imaging. Result of intra-rater ICC also showed both 3D imaging and 2D photography had good reliability in all 4 nasal angles. Similar to those of inter-rater ICC, nasofacial angular parameter showed statistically significant differences between 3D imaging and 2D photography. In terms of reliability, both 2D and 3D showed appropriate anthropometric results and considering its own advantage, each methods can be used complementarily.

## Introduction

Nasal shape assessment in the field of facial plastic surgery is important because it provides aesthetic information objectively^1^. For this reason, various techniques have been attempted for evaluating external nose with the development of photograph technology^1^. Photography for evaluating an external nose is based on measuring landmarks in photographs taken from different angles. Although it has been an ideal instrument for facial analysis for half a century, it has some limitations produced by changing position and lighting. In addition, it is insufficient to describe three-dimensional (3D) structures in two dimensions (2D)^1,2^. Through advances in technology, numerous techniques of facial imaging using 3D images have been attempted^2^. 3D analysis is important for understanding normal craniofacial structure, preoperative planning, and postoperative assessment^3^. However, a standard ideal method for analyzing the face has not been established yet^2^. Additionally, previous studies have rarely reported reliability estimates for both anthropometric techniques depending on nasal angular parameters, leading to a great concern as to which technique will yield adequate results for inter-rater and intra-rater (test–retest) reliability and whether 3D assessment is superior to conventional 2D assessment. Therefore, the objective of this study was to compared the reliability and reproducibility of two facial anthropometric methods, 3D imaging and conventional 2D photography.

## Materials and methods

### Subjects

This study was carried out at Kosin University Gospel Hospital, Busan, South Korea, between November 2019 and June 2020. Subjects had no history of nasal trauma or surgical treatment between imaging procedures. Any images with poor quality were excluded. The Kosin University Institutional Review Board approved the study design (2021-02-022). Thirty healthy volunteers aged 20–29 years were included in this study. Participants were informed about the procedures. They were provided informed consent for documentation, study participation and online open-access publication of images. All methods were carried out in accordance with relevant guidelines and regulations.

### Standardization

**Figure 1:**
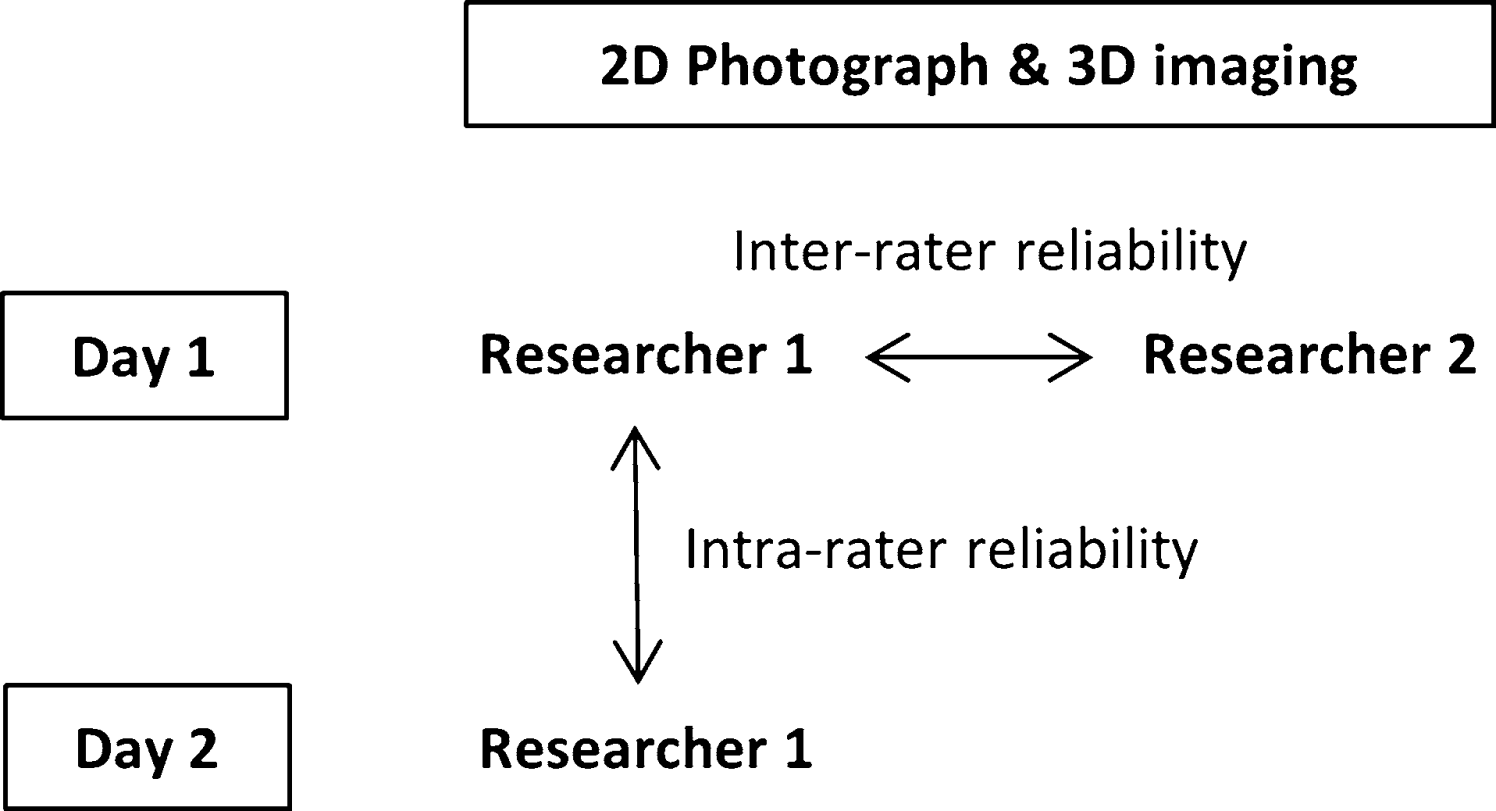
Schematic diagram of the study design. On the first day, 2D photograph and 3D imaging were taken for each subject by two reserchers. On another day, both 2D, 3D images were taken by researcher 1.

To evaluate intra-rater reliability, 2D photograph and 3D digital images were taken over two different days for each subject by the same researcher. To evaluate inter-rater reliability, another researcher took 2D photographs and 3D digital images for each subject on the first day (Fig. 1). All images were taken with the same instruments and the same standardization which were described as follows. 2D photography was performed at the same place (blue background). At a distance of two meters from the camera, subjects seated in a fixed position. They were asked to gaze directly at fixed points. The height was adjusted individually. For right lateral photographs, subjects were seated in such a way that the head along with the visual axis aligned parallel to the floor of the room with respect to the Frankfort horizontal plane (a line from the most superior point of the external auditory canal to most inferior point of the infraorbital rim). Glasses were removed with eyes set at the same level and open.

### Landmarks

**Figure 2:**
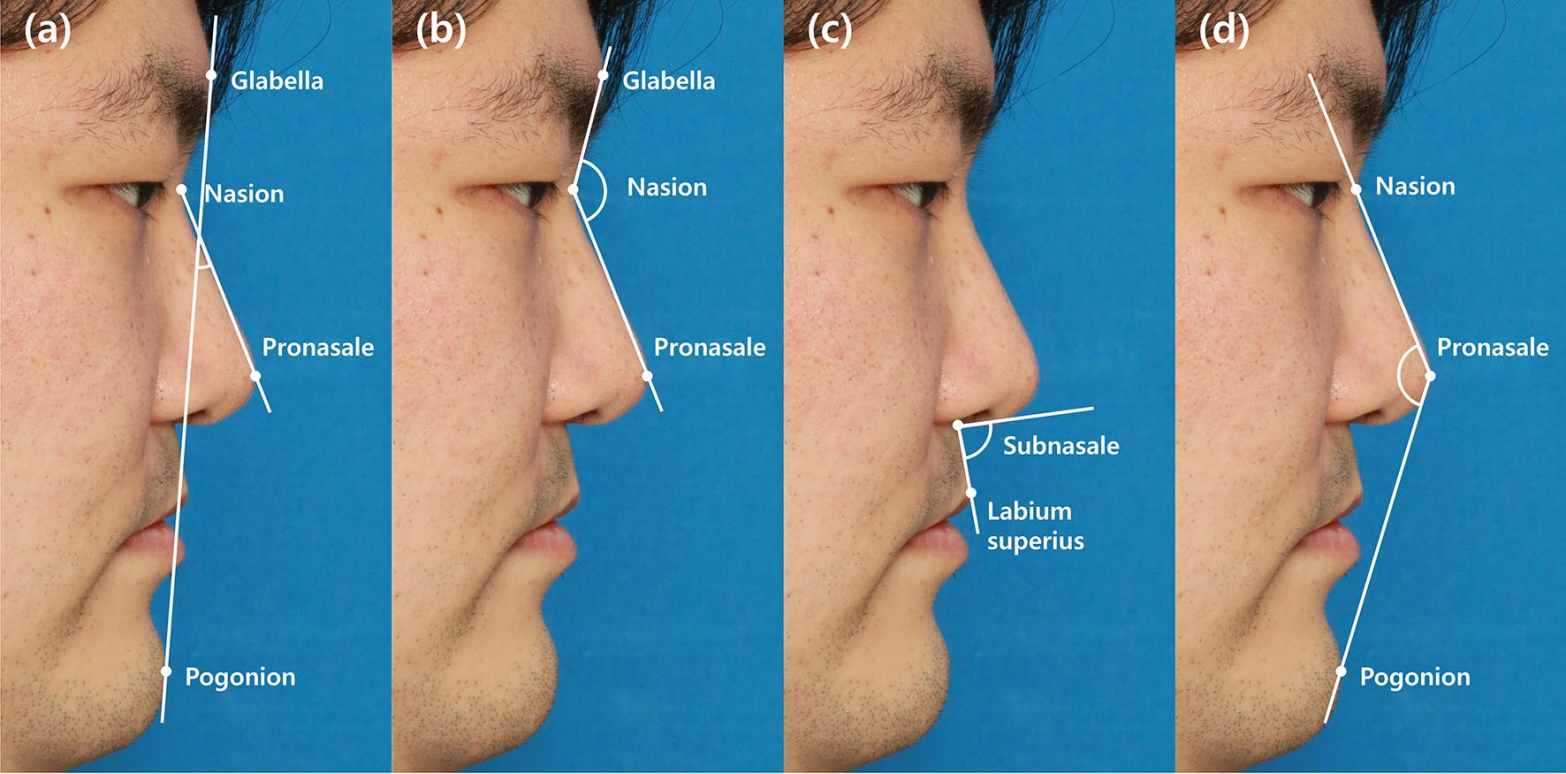
Landmarks on lateral view of 2D photograph. Four angular components were derived as follows. (**a**) Nasofacial angle: nasion-pronasale line intersecting with glabella-pogonion line. (**b**) Nasofrontal angle: glabella-nasion-pronasale. (**c**) Nasolabial angle: subnasale-columella line intersecting with subnasale-labium superius line. (**d**) Nasomental angle: nasion-pronasale-pogonion.

Four angular parameters (nasofrontal angle, nasofacial angle, nasolabial angle, nasomental angle) from image were taken by the same researcher. Six profile landmarks (glabella, nasion, pronasale, subnasale, labium superius, and pogonion) were used. Glabella was defined as the most prominent point of the forehead in the midline between eyebrows. Nasion was the deepest point of the nasal bridge on the intersection between the midline of the nasal root and the nasofrontal suture. Pronasale was the most prominent point of the nasal tip. Subnasale was the deepest point at the junction of the base of the columella and the upper lip in the midline. Labium superius was the most prominent midpoint on the vermilion line of the upper lip. Pogonion was the most prominent point of the chin in the midline. Each landmark (glabella, nasion, pronasale, subnasale, labium superius, and pogonion) was marked and connected for angle measurement. To measure the nasofrontal angle, the nasofacial angle, the nasolabial angle, and the nasomental angle, glabella-nasion-pronasale, or the nasion-pronasale line intersecting with the pronasale-pogonion line, or subnasale-columella line intersecting with subnasale-labium superius line, nasion-pronasale-pogonion was connected, respectively (Fig. 2)^4,5^.

### Two-dimensional assessment

2D photographs were taken four times for each subject using a digital camera (Canon EOS 70D., Canon, Tokyo, Japan). Adobe Photoshop CS5 (Adobe Systems Inc., San Jose, CA, USA) was used to edit landmark locations and to measure external nasal angles. To evaluate inter-rater reliabilities, two raters took photographs for each subject. To evaluate the test–retest reliability, one rater took photographs twice over at least two days (Fig. 1). The rater was trained to shoot the same pose with the same camera at the same distance for standardization. Front, left and right lateral photographs were taken, but only right lateral images were selected for angular measurement. Obtained photographs were evaluated for angle and distance according to each index by an observer.

### Three-dimensional assessment

**Figure 3:**
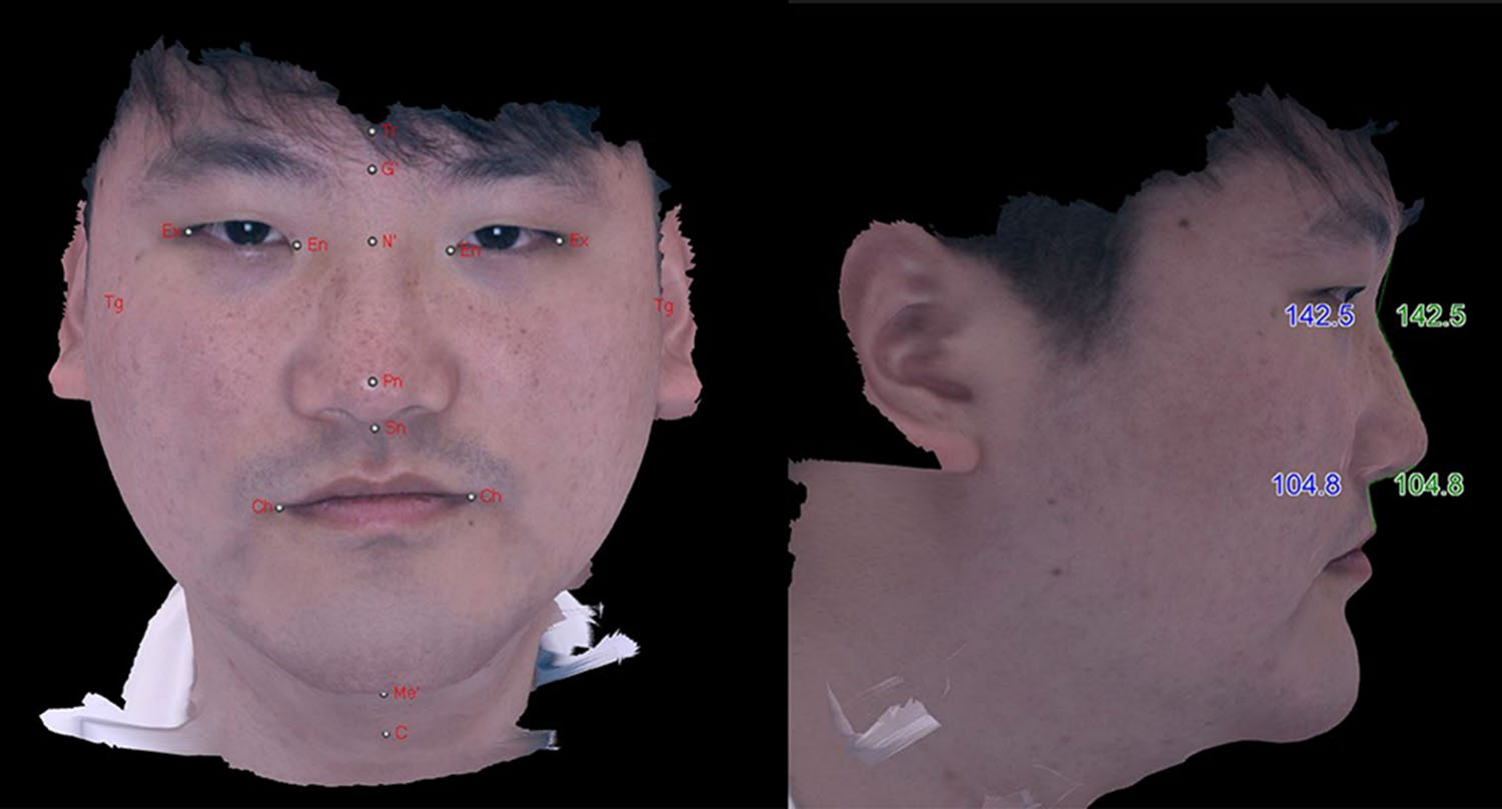
Reconstructed image of 3D scanning. Each landmark on the rendered image were detected and angular components were measured automatically through the landmarks. The landmarks can be readjusted by researchers.

3D digital images were taken the same condition as 2D photograph using a Morpheus 3D scanner (Morpheus Co., Ltd., Seongnam City, Gyunggi-do, Korea). Participants underwent 3D imaging with the forehead and ear neck visible, sitting in a neutral head position about 500 mm from the lens of a 3D scanner. The scanning was performed from frontal, left, and right oblique views at the same brightness. Images taken with the scanner were automatically merged and reconstructed into 3-dimension. In the rendered image, each landmark was automatically detected and located on the surface of the rendered image, and then adjusted by the rater when it was misplaced from the definition of the landmark (Fig. 3).

### Statistical analysis

Reliability reflects the degree of agreement between repeated measures. It is also described as precision, repeatability, or reproducibility. Analysis was done using statistical software; SPSS version 11.0.1 (SPSS, Inc, Chicago, IL, USA). Intraclass correlation coefficient (ICC) was calculated to evaluate the reliability^6^. ICC < 0.5, = 0.5 to 0.75, and ≥ 0.75 indicated poor, satisfactory, and good to excellent agreement, respectively. Using a Welch’s *t* test, reliability of 3D imaging and 2D photography were compared.

## Results

**Figure 4:**
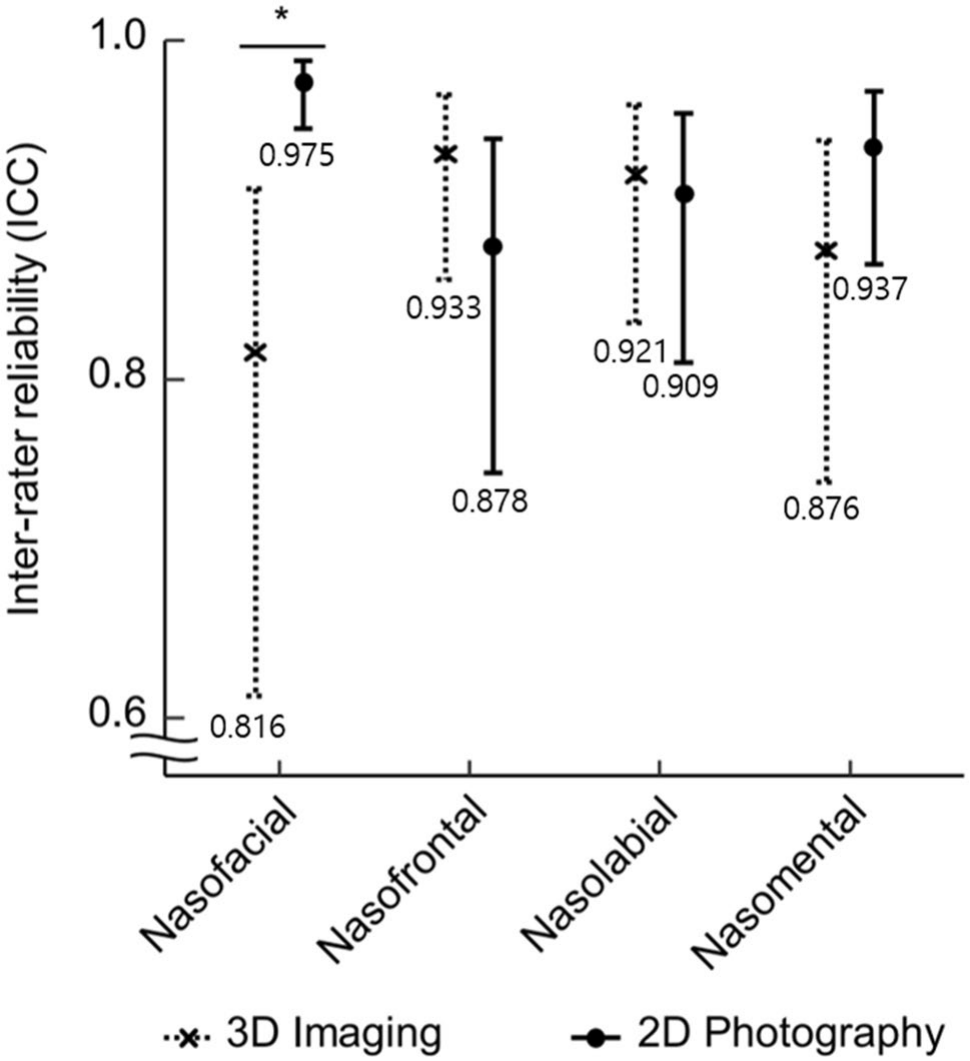
Comparison of inter-rater reliability on 3D imaging and 2D photograph (crosses and dots represented ICC, error bars represented 95% CI) showed that inter-rater reliability had significant difference for nasofacial angle while other angular parameters had no statistically significant difference between the two techniques (*p < 0.001). The value under error bars represented ICC value.

Estimated intra-rater and inter-rater ICC are shown in Table 1. Inter-rater and intra-rater ICC and 95% CIs of four angular variables with each technique were taken. Regarding the inter-rater reliability, both 3D imaging and 2D photography showed significant reliable results for all four angular variables. All inter- and intra-rater ICCs were ≥ 0.75, indicating excellent agreement and good reliability (Fig. 4).

**Table 1:** Results of nasal angle range and inter-rater and intra-rater reliability of 3D imaging and 2D photography based on intraclass correlation coefficient (ICC) and 95% CI.

Angles	Reliability	3D imaging			2D Photography			*p* Value
		Range (°)	ICC	95% CI	Range (°)	ICC	95% CI	
Nasofacial	Inter-rater	25.8–53.5	0.816	0.61–0.91	26.4–42.6	0.975	0.95–0.99	0.001
	Intra-rater		0.836	0.65–0.92		0.956	0.91–0.98	0.008
Nasofrontal	Inter-rater	123.4–162.6	0.933	0.86–0.97	120.7–155.2	0.878	0.74–0.94	0.142
	Intra-rater		0.960	0.92–0.98		0.925	0.84–0.96	0.125
Nasolabial	Inter-rater	86.9–132.1	0.921	0.83–0.96	76.6–123.3	0.909	0.81–0.96	0.738
	Intra-rater		0.919	0.83–0.96		0.935	0.86–0.97	0.580
Nasomental	Inter-rater	109.8–141.5	0.876	0.74–0.94	122–153.6	0.937	0.87–0.97	0.100
	Intra-rater		0.899	0.79–0.95		0.934	0.86–0.97	0.277

For all ICC values, *p* value < 0.001.

For inter-rater ICC, 2D photography showed higher values than 3D imaging for nasofacial and nasomental angles (nasofacial angle in 2D photography: 0.975, 95% CI: 0.95–0.99; nasomental angle in 2D photography: 0.937, 95% CI: 0.87–0.97) (Fig. 4). Especially, nasofacial angular inter-rater ICCs showed significantly differences between 2D photography and 3D imaging (2D photography vs. 3D imaging: 0.975 vs. 0.816, *p* value < 0.001). The other two angular parameters, nasofrontal and nasolabial angles, showed higher ICCs without showing statistically significant differences between 3D imaging and 2D photography (nasofrontal angle in 3D imaging: 0.933, 95% CI: 0.86–0.97; nasofrontal angle in 2D photography: 0.878, 95% CI: 0.74–094, *p* value < 0.142; nasolabial angle in 3D imaging: 0.921, 95% CI: 0.83–0.96; nasolabial angle in 2D photography: 0.909, 95% CI: 0.81–0.96, *p* value < 0.738).

**Figure 5:**
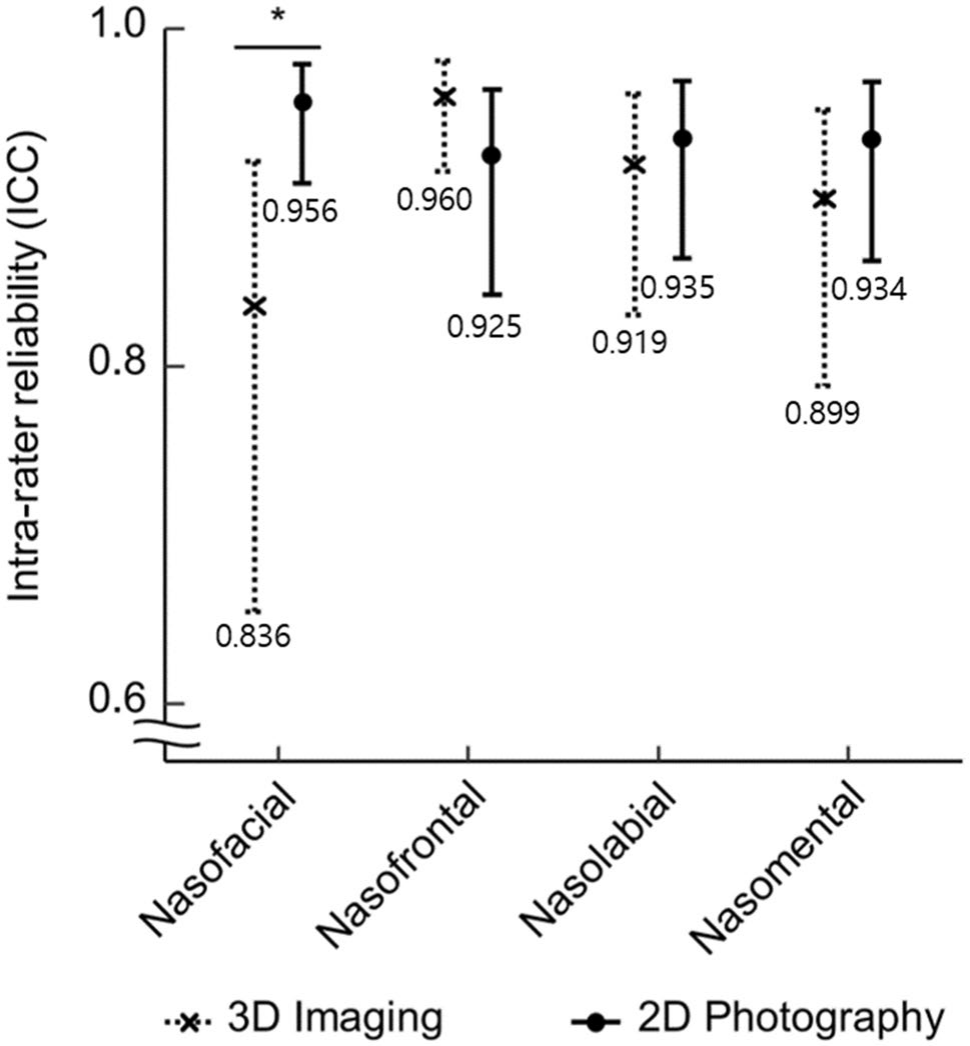
Comparison of intra-rater reliability of 3D imaging and 2D photograph (Crosses and Dots represented ICC, error bars represented 95% CI), showed significant difference in intra-rater reliability for the nasofacial angle (*p < 0.008). The value under error bars represented ICC value.

For the intra-rater ICC, all estimates showed excellent reliability in four angular variables with both imaging technique. For the nasofacial/nasolabial/nasomental angular intra-rater reliability, 2D photography showed superiority over 3D imaging (nasofacial/nasolabial/nasomental angle in 2D photography: 0.956/0.935/0.934) (Fig. 5). Regarding the nasofrontal angular intra-rater reliability, 2D photography showed lower reliability than 3D imaging (nasofrontal angle in 3D imaging: 0.960, 95% CI: 0.92–0.98). For the nasofacial angular parameter, 2D photography showed significant higher reliability than 3D imaging (2D photography vs. 3D imaging: 0.956 vs. 0.836, *p* value < 0.008).

## Discussion

With wide spread of facial plastic reconstructive surgery, normative anthropometric database plays an important role in diagnosis such as determining the location, size, and the extent of deformity. It also provides essential information in determining a treatment plan, and playing an important role in showing the difference between pre- and post-operative data^7,8^. Conventionally, the nasal profile is measured through a photograph, which is done manually by landmarks. Therefore, there might be errors in the repeated measurement. In addition, it is a time-consuming work^7^. It also has limitation in illustrating 3D structures because a 2D form cannot evaluate facial depth or shape^9^. 2D photographs have another limitation in that results may vary depending on head position or changes of setting^2^. To overcome these limitations, various techniques with a 3D anthropometric approach have been introduced. For rhinoplasty, 3D imaging technique plays an important role, and is widely used by plastic surgeons. However, there has been no universally accepted platform, nowadays^2,10^.

There have been several studies about three-dimensional imaging. Van Heerbeek et al.^11^ have evaluated whether 3D imaging is capable of and useful for measuring and objectifying rhinoplasty results. According to Zogheib et al.^12^, 3D imaging could enable angular measurement of human face as much as a photograph. Through these current studies, 3D imaging could be used to study whether surgical techniques have the desired effect on the nose.

In this study, we investigated reproducibility of conventional 2D photography and 3D imaging techniques based on their inter- and intra-rater reliability for four angular parameters. Results of this study revealed that all inter- and intra-rater reliability of both imaging techniques showed intraclass correlation coefficient values higher than 0.75, meaning that both 2D photograph and 3D imaging had excellent reproducibility. Hence, both conventional 2D photography and 3D imaging are feasible anthropometric methods for measuring external nasal angles.

The authors also compared reliability of both imaging methods. Comparing the reliability of nasal angular parameters, we found that 2D photography showed significantly higher reliability than 3D imaging for the nasofacial angular parameter. For the other three angular parameters, they showed no significant difference. we infer that error occurs when setting and correcting the landmarks on 3D images because correcting landmarks on 3D structure were more complicated than 2D image. Because, considering the planes of space in three dimension(X, Y, Z), landmarks can be placed at more various locations in the 3D image, than in 2D photograph which can be placed on the surface of the line^13^. Since it is difficult to place landmarks on the lateral view in 3D image, errors are more likely to occur when inexperienced researcher set landmarks^13^. In this study, nasofacial angle is set as a component of a glabella-pogonion line consisting of less reproducible landmarks, glabella and pogonion, suggesting that more errors may occur when placing landmark in a 3D image than in a 2D image^13,14^.

Aside from this point, 3D assessment of facial anthropometry still has its own advantages. Lekakis et al. have described several advantage of 3D imaging, stating that 3D images offer an excellent patient teaching tool^1^. Additionally, Honrado and Larrabee^9^ have mentioned that 3D imaging will help surgeons perform surgical planning and assess the outcome with increased understanding of structures.

As a limitation of this study, we didn’t take into account the experience of the researcher in setting or correcting the landmark, which means if experienced researchers performed this procedure, the reliability might be different. Another limitation was that our study was designed only as an angular study of external nose because there was no comparable value of real nasal anthropometric parameter.

In summary, 2D and 3D imaging showed high reliability for all angular parameters, which did not show a significant difference in reliability except for the nasofacial angle. The authors thought that there might be technical errors in the assessment of nasofacial angular parameters of 3D imaging. However, 3D imaging has its own advantages that is easy to explain the images to the patients and is easy to get the its anthropometric results repeatedly. The authors suggest that both 2D photography and 3D imaging in anthropometric technique for nasal angles are reliable and complementary each other. Further studies and technological development are needed to improve the accuracy and reliability of the results.

## Conclusion

This study showed that both 3D imaging and 2D photography had good inter- and intra -rater reliability for four nasal angles, and complementary to each other for measuring external nasal anthropometry.
